# Association of red cell distribution width with pulmonary arterial hypertension in patients with mixed connective tissue disease

**DOI:** 10.1186/s12890-023-02597-z

**Published:** 2023-08-14

**Authors:** Yansheng Jin, Guanjun Guo, Chun Wang, Bo Jiang

**Affiliations:** 1 Department of Rheumatology and Immunology, The Affiliated Drum Tower Hospital of Nanjing University Medical School, 321 Zhongshan Road, Nanjing, Jiangsu Province 210008 China; 2 Department of Rheumatology and Immunology, Suzhou Wuzhong People’s Hospital, 61 Dongwu North Road, Suzhou, Jiangsu Province 215128 China; 3 Department of Cardiology, Cardiac Function Room, The Affiliated Drum Tower Hospital of Nanjing University Medical School, 321 Zhongshan Road, Nanjing, Jiangsu Province 210008 China

**Keywords:** Red cell distribution width, Pulmonary arterial hypertension, Mixed connective tissue disease

## Abstract

**Background:**

Pulmonary arterial hypertension (PAH) is a severe complication of mixed connective tissue disease (MCTD) and contributes to increased morbidity and mortality. Still, the demographic characteristics and risk factors of PAH in MCTD remain poorly understood. This study explored risk factors for PAH development in MCTD.

**Methods:**

Data from patients with MCTD and PAH hospitalized from May 2009 to December 2022 in a single center were collected and compared with patients with MCTD without PAH. The variables were analyzed by logistic regression to identify the factors associated with PAH in patients with MCTD. The receiver-operating characteristic (ROC) curve was used to assess the diagnostic value of the identified factors.

**Results:**

Finally, 119 patients with MCTD were included; 46 had PAH. The mean age at PAH onset and diagnosis was 38.9 ± 13.4 and 39.9 ± 13.7 years, respectively. The median pulmonary arterial systolic pressure (PASP) was 67.0 mmHg. The median brain natriuretic peptide (BNP) level was 180.0 pg/ml at PAH diagnosis. Red cell distribution width (RDW) (OR: 2.128; 95% confidence interval: 1.497–3.026; *P* < 0.001) was associated with PAH in patients with MCTD. There was a positive correlation between RDW and PASP (r = 0.716, *P* < 0.001). At a cutoff of 15.2%, RDW had the best sensitivity (80.4%) and specificity (82.2%) for PAH.

**Conclusion:**

RDW may serve as a sensitive index to predict PAH in patients with MCTD.

**Supplementary Information:**

The online version contains supplementary material available at 10.1186/s12890-023-02597-z.

## Background

Mixed connective tissue disease (MCTD) is one of the most complicated autoimmune diseases and involves various overlapping symptoms [1]. It is characterized by diverse clinical manifestations of systemic sclerosis (SSc), systemic lupus erythematosus (SLE), polymyositis or dermatomyositis (PM/DM), and rheumatoid arthritis (RA), which can also affect multiple organs, including the pulmonary system [2]. Pulmonary involvements, especially pulmonary arterial hypertension (PAH), are devastating complications among patients with MCTD, resulting in increased morbidity and mortality [3–5]. The prevalence of PAH is 2-24% in patients with MCTD, depending on the measurement methods used [3]. So far, little knowledge is available about the clinical characteristics of PAH patients associated with the MCTD population. The present study aimed to explore the factors associated with PAH in patients with MCTD and determine the diagnostic value of the identified factors.

## Methods

### Patients and diagnostic criteria

Patients with MCTD hospitalized in the rheumatology and immunology department of the affiliated Drum Tower Hospital of Nanjing University Medical School between May 2009 and December 2022 were included in this study. The exclusion criteria were (a) < 18 years of age, (b) complicated with other connective tissue diseases (CTD) such as SSc, SLE, or primary Sjögren’s syndrome (pSS), (c) PAH caused by cardiac structural lesions, such as left heart disease or valvular heart disease, (d) with severe pulmonary diseases resulted from lung cancer or chronic obstructive pulmonary disease, or (e) PAH due to liver cirrhosis, pulmonary venous occlusive disease, or chronic thromboembolism. All patients met the established MCTD criteria [6].

PAH was defined according to the 2022 ESC/ERS guidelines, using tricuspid regurgitation velocity (TRV) > 2.8 m/s [7]. The initial diagnosis or screening of PAH was established based on transthoracic echocardiography (TTE)-estimated resting pulmonary arterial systolic pressure (PASP) ≥ 36 mmHg [7], while those with PASP < 36 mmHg and without any other echocardiographic indicators suggesting PAH were included in the MCTD-non-PAH group. Written informed consent was obtained from each subject following a detailed explanation of the objectives and protocol of the study. The study was conducted in accordance with the Declaration of Helsinki and was approved by the ethics committee of Nanjing Drum Tower Hospital (No.2020-093-01).

### Data collection

Demographic data, including sex, age at MCTD onset, and disease duration, height, weight, body mass index (BMI), body surface area (BSA), and disease activity were collected. In addition, age at PAH onset and diagnosis was also included for patients with PAH, in which the former was defined as the time when the initial symptoms appeared, while the latter was when TTE examination showed abnormalities. The nonspecific symptoms were recorded, such as Raynaud’s phenomenon, sclerodactyly, serositis, limb edema, fatigue, arthralgia, and chest tightness. The World Health Organization functional classification (WHO-FC) was independently evaluated by two authors (YJ and BJ) for each patient with PAH.

TTE was performed using a Philips IE Elite machine. The included patients were all examined by TTE to evaluate the likelihood of PAH. The peak PASP was calculated using the simplified Bernoulli equation (PASP = 4 tricuspid regurgitation^2^ + right atrial pressure) [8]. The other parameters based on TTE were included. Autoantibodies were routinely tested. Other laboratory data, including complete blood count, uric acid (UA), blood lipid analysis, C-reactive protein (CRP), erythrocyte sedimentation rate (ESR), thyroid function test, complement, immunoglobulin, blood coagulation function test, and brain natriuretic peptide (BNP) levels were also documented. The hemoglobin and mean corpuscular volume (MCV) were used to evaluate the iron deficiency. The Systemic Lupus Erythematosus Disease Activity Index 2000 (SLEDAI-2 K) was used to evaluate disease activity [9].

### Statistical analysis

Analyses were done using SPSS 19.0 (IBM, Armonk, NY, USA) and MedCalc 19.1 (MedCalc, Ostend, Belgium). The variables were presented as means ± standard deviations ± or medians with interquartile ranges. Student’s t-test or Mann-Whitney U-test was used for comparisons. The categorical variables were presented as n (%) and analyzed using the chi-square test. The correlations between two continuous variables were determined using Spearman’s correlation coefficient. Factors associated with PAH in the univariable logistic regression analyses were evaluated using multivariable logistic regression analysis. The receiver operating characteristic (ROC) curve with the area under the curves (AUC) was plotted to investigate the diagnostic value of the factor. The optimal cutoff was assessed using Youden’s index in ROC analysis. Survival was analyzed using the Kaplan-Meier method and the log-rank test. *P <* 0.05 was considered statistically significant.

## Results

### Characteristics of the patients

This study included 119 patients with MCTD, among whom 46 were diagnosed with PAH (MCTD-PAH), and 73 did not have PAH (MCTD-non-PAH). The demographics of the patients are shown in Table 1. The female-to-male ratio in the MCTD-PAH and MCTD-non-PAH groups were 44:2 and 69:4, respectively (*P* > 0.05). The mean age at MCTD diagnosis in the MCTD-PAH and MCTD-non-PAH groups was 37.8 ± 13.4 years vs. 39.8 ± 12.2 years (*P* > 0.05), while the mean age at MCTD onset in MCTD-PAH and MCTD-non-PAH groups were 35.1 ± 12.8 years vs. 36.5 ± 11.8 years (*P* > 0.05). The mean age at PAH onset was 38.9 ± 13.4 years, while PAH diagnosis was 39.9 ± 13.7 years, respectively. Speculatively, PAH diagnosis according to TTE was found to be 1.0 ± 1.6 years’ delayed from PAH onset. The height, weight, BMI, and BSA were comparable between two groups (all *P* > 0.05). Six, 33, and seven patients had low-risk, middle-risk, and high-risk PAH. They were 36.7 ± 11.9, 46.4 ± 12.9, and 42.4 ± 14.5 years of age, respectively, and there were six, 31, and seven females, respectively.

**Table 1:** Baseline demographics, laboratory data, and autoantibody profiles between MCTD-PAH and MCTD-non-PAH patients

Variable	MCTD-PAH group (n = 46)	MCTD-non-PAH group (n = 73)	Statistical values	*P*	
**Demographics**					
Sex [F/M, n (%)]	44 (95.7)/2 (4.4)	69 (94.5)/4 (5.5)	0.000	> 0.999	
Age at MCTD onset (years, $\bar x$*±s*)	35.1 ± 12.8	36.5 ± 11.8	-0.603	0.547	
Age at MCTD diagnosis (years, $\overline{ x}$*±s*)	37.8 ± 13.4	39.8 ± 12.2	-0.831	0.408	
Age at PAH onset (years, $\bar x$*±s*)	38.9 ± 13.4				
Age at PAH diagnosis (years, $\bar x$*±s*)	39.9 ± 13.7				
Height [m, M (P25-P75)]	1.61 (1.58–1.64)	1.61 (1.58–1.63)	-0.008	0.993	
Weight (kg, $\bar x$*±s*)	60.76 ± 4.17	59.43 ± 3.50	1.874	0.063	
BMI (kg/m^2^, $\bar x$*±s*)	23.49 ± 1.49	22.99 ± 1.32	1.875	0.064	
BSA (m^2^, $\bar x$*±s*)	1.72 ± 0.06	1.71 ± 0.05	1.545	0.125	
**Clinical manifestations** [n(%)]					
SLEDAI-2 K Activity			-0.052	0.958	
Almost inactive	20	31			
Mild	15	25			
Moderate	8	12			
Severe	3	5			
SLEDAI-2 K scores	5.00 (1.00-9.25)	5.00 (4.00–9.00)	-0.907	0.364	
Raynaud’s phenomenon	42 (91.3)	67 (91.8)	0.000	> 0.999	
Sclerodactyly	15 (32.6)	17 (23.3)	1.247	0.264	
Serositis	29 (63.0)	20 (27.4)	14.803	*<* 0.001	
Limbs edema	8 (17.4)	7 (9.6)	1.559	0.212	
Fatigue	26 (56.5)	36 (49.3)	0.587	0.443	
Arthralgia	31 (67.4)	43 (58.9)	0.864	0.353	
Chest tightness	35 (76.1)	24 (32.9)	21.076	*<* 0.001	
**Laboratory profiles**					
WBC (×10^9^/L, $\bar x$*±s*)	5.9 ± 2.8	7.6 ± 8.0	-1.426	0.156	
RBC [×10^12^/L, M (P25-P75)]	4.3(3.8–4.8)	4.3(3.8–4.5)	-0.669	0.504	
PLT (×10^9^/L, $\bar x$*±s*)	185.0 ± 84.9	206.7 ± 101.8	-1.207	0.230	
HGB (g/L, $\bar x$*±s*)	124.6 ± 11.5	124.5 ± 12.1	0.079	0.937	
MCV (fL, $\bar x$*±s*)	87.1 ± 3.3	86.4 ± 3.3	1.260	0.210	
RDW [%, $\bar x$*±s*]	18.2 ± 3.4	13.8 ± 1.7	8.174	*<* 0.001	
UA (µmol/L, $\bar x$*±s*)	320.5 ± 132.1	270.8 ± 97.9	2.200	**0.031**	
CRP [mg/L, M (P25-P75)]	4.2 (2.5–11.3)	4.1 (2.5–8.9)	-0.612	0.541	
ESR [mm/hr, M (P25-P75)]	29.5 (13.5–55.0)	29.0 (12.0-43.5)	-0.972	0.331	
TSH [mU/L, M (P25-P75)]	2.6 (1.1–4.9)	1.3 (0.8–2.7)	-2.423	**0.015**	
FT3 (pmol/L, $\bar x$*±s*)	3.0 ± 1.2	3.5 ± 1.0	-3.130	**0.002**	
FT (pmol/L, $\bar x$*±s*)	14.9 ± 3.3	15.4 ± 3.0	-0.887	0.377	
C3 (g/L, $\bar x$*±s*)	0.9 ± 0.3	1.0 ± 0.3	-1.648	0.102	
C4 (g/L, $\bar x$*±s*)	0.2 ± 0.1	0.4 ± 0.8	-1.852	0.067	
IgA [g/L, M (P25-P75)]	2.1 (1.5–3.3)	2.7 (2.0-3.4)	-1.839	0.066	
IgG [g/L, M (P25-P75)]	17.0(12.1–27.5)	16.7 (13.1–20.0)	-0.854	0.393	
IgM (g/L, $\bar x$*±s*)	1.3 ± 0.7	1.4 ± 0.9	-0.534	0.594	
D-Dimer [mg/L, M (P25-P75)]	0.5 (0.2–1.4)	0.5 (0.3–0.8)	-1.561	0.118	
Fg [g/L, M (P25-P75)]	2.6 (2.3–2.9)	2.6 (2.3-3.0)	-0.014	0.989	
PT [s, M (P25-P75)]	18.5 (17.5–19.5)	18.9 (18.4–19.6)	-1.177	0.239	
APTT (s, $\bar x$*±s*)	27.8 ± 4.9	28.3 ± 5.0	-0.526	0.600	
Autoantibody profiles [positive number, n(%)]					
Nuclear speckled	38 (82.6)	54 (74.0)	1.200	0.273	
Nuclear homogeneous	1 (2.2)	1 (1.4)	—	1.00	
Nuclear membrane	1 (2.2)	1 (1.4)	—	1.00	
Nucleolar	1 (2.2)	1 (1.4)	—	1.00	
Nuclear centromeric	1 (2.2)	4 (5.5)	0.165	0.685	
Cytoplasmic	7 (15.2)	12 (16.4)	0.031	0.859	
U1-RNP	35 (76.1)	48 (65.8)	1.428	0.232	
Sm	3 (6.5)	6 (8.2)	—	1.00	
SS-A	14 (30.4)	13 (17.8)	2.565	0.109	
RO52	10 (21.7)	12 (16.4)	0.526	0.468	
SS-B	5 (10.9)	3 (4.1)	1.120	0.290	
SCL70	1 (2.2)	1 (1.4)	—	1.00	
Jo-1	1 (2.2)	2 (2.7)	—	1.00	
dsDNA	1 (2.2)	1 (1.4)	—	1.00	

F, femal; M, male; BMI, body mass index; BSA, body surface area; SLEDAI-2 K, Systemic Lupus Erythematosus Disease Activity Index 2000; WBC, white blood cell; RBC, red blood cell; PLT, platelet; HGB, hemoglobulin; MCV, mean corpuscular volume; RDW, red cell distribution width; UA, uric acid; CRP, C-reactive prolein; ESR, rythrocyte sedimentation rate; TSH, thyroid stimulating homone; FT3, free triiodothymnine; FT, free thyroxine; C, complement; Ig, immunoglobulin; Fg, fibrinogen; PT, prothmmbin time; APTT, activated partial thmmboplastin time; WHO FC, World Health Organization functional class; U1-RNP, U1 ribonucleoprotein; Sm, Smith; SS-A, Sjogren’s-syndrome-related antigen A/anti-Ro; SS-B, Sjogren’s-syndrome-related antigen B/anti-La; SCL-70, topoisomerase 1; Jo-1, histidyl-tRNA synthetase; ds, double-stranded

As for the symptoms (Table 1), serositis (63.0% vs. 27.4%, *P* < 0.001) and chest tightness (76.1% vs. 32.9%, *P <* 0.001) in the MCTD-PAH group were significantly more common than in the MCTD-non-PAH group. Raynaud’s phenomenon, sclerodactyly, limb edema, fatigue, disease activity and arthralgia showed no differences between the two groups.

### Laboratory findings

Compared with the MCTD-non-PAH group, the patients in the MCTD-PAH group displayed larger red cell distribution width (RDW) [(18.2 ± 3.4) % vs. (13.8 ± 1.7) %, *P <* 0.001] and higher UA levels [(320.5 ± 132.1) vs. (270.8 ± 97.9) µmol/L, *P* < 0.05] (Table 1). There were no significant differences in hemoglobin and MCV between the two groups (both *P* > 0.05). Interestingly, serum free triiodothyronine (FT3) was significantly decreased in MCTD-PAH patients [(3.0 ± 1.2) vs. (3.5 ± 1.0) pmol/L, *P* < 0.01], while thyroid stimulating hormone (TSH) was significantly higher in the MCTD-PAH group [2.6 (1.1–4.9) vs. 1.3 (0.8–2.7) mU/L, *P* < 0.05]. The autoantibody was not different between the MCTD-PAH and MCTD-non-PAH groups (Table 1). The other laboratory test values were comparable between the two groups (*P >* 0.05).

### Echocardiographic findings

The echocardiagram findings are summarized in Table 2. Compared with the MCTD-non-PAH group, the MCTD-PAH group had significantly lower left ventricular diastolic dimension (LVDd) [(4.5 ± 0.5) vs. (4.7 ± 0.4) cm, *P <* 0.01], pulmonary artery velocity (PA) [(0.8 ± 0.1) vs. (0.9 ± 0.2) m/s, *P <* 0.05], left atrial diameter (LAD) [(3.5 ± 0.7) vs. (4.4 ± 0.6) cm, *P <* 0.05], and left ventricle ejection fraction (LVEF) [59.0 (58.0-60.3)% vs. 61.0 (60.0-61.5)%, *P <* 0.01], respectively. Besides, TTE confirmed pericardial effusion in 37.0% of the MCTD-PAH patients and 32.6% of the MCTD-non-PAH patients (*P* < 0.05). Other indexes, including interventricular septum thickness diastolic (IVSTd), left ventricular posterior wall thickness at end-diastole (LVPWTd), aorta diameter (AoD), left ventricular end-systolic dimension (LVDs), and mitral e peak/mitral a peak (E/A), were similar between the two groups (*P >* 0.05).

**Table 2:** Comparisons of echocardiographic findings, BNP, and WHO Fc between MCTD-PAH and MCTD-non-PAH patients

Variable	MCTD-PAH group (n = 46)	MCTD-non-PAH group (n = 73)	Statistical values	*P*
IVSTd [cm, M (P25-P75)]	0.8 (0.8–0.9)	0.8 (0.8–0.9)	-1.373	0.170
LVPWTd [cm, M (P25-P75)]	0.8 (0.8–0.9)	0.80 (0.8–0.9)	-0.969	0.333
LVEF [%, M (P25-P75)]	59.0 (58.0-60.3)	61.0 (60.0-61.5)	-2.970	**0.003**
LVDd (cm, $\bar x$*±s*)	4.5 ± 0.5	4.7 ± 0.4	-3.130	**0.003**
AoD [cm, M (P25-P75)]	2.8 (2.8–3.1)	3.0 (2.7–3.3)	-0.757	0.449
PA (m/s, $\bar x$*±s*)	0.8 ± 0.1	0.9 ± 0.2	-2.317	**0.022**
LVDs [cm, M (P25-P75)]	3.1 (2.8–3.3)	3.2 (3.0-3.3)	-1.737	0.082
LAD [cm, M (P25-P75)]	3.5 ± 0.7	4.4 ± 0.6	-2.079	**0.038**
E/A [M (P25-P75)]	0.9 (0.8–1.4)	1.2 (0.8–1.4)	-0.764	0.445
Pericardial effusion [P/N, n (%)]	17 (37.0)/29 (63.0)	15 (32.6)/58 (79.5)	3.865	**0.049**
PASP [mmHg, M (P25-P75)]	67.0 (48.5–90.0)	< 36	**—**	
BNP [pg/mL, M (P25-P75)]	180.0 (63.8-478.5)	57.3 (22.8–112.0)	-5.621	< 0.001
WHO Fc [I-II/III-IV, n (%)]	25 (54.4)/21 (45.7)	72 (98.6)/1 (1.4)	36.719	< 0.001

IVSTd, interventricular septum thickness diastolic; LVPWTd, left ventricular posterior wall thickness at end-diastole; LVEF, left ventricle ejection fraction; LVDd, left ventricular diastolic dimension; AoD, aorta diameter; PA, pulmonary artery velocity; LVDs, left ventricular end-systolic dimension; LAD, left atrial diameter; E, mitral e peak; A, mitral a peak; PASP, pulmonary arterial systolic pressure; BNP, brain natriuretic peptide; WHO FC, World Health Organization functional class; P, positive; N, negative

As shown in Table 2, many patients with PAH had severe cardiac involvement at PAH diagnosis, with a median PASP of 67.0 (interquartile range 48.5–90.0) mmHg and a median level of BNP of 180.0 (interquartile range 63.8-478.5) pg/mL. The BNP levels were higher in the MCTD-PAH group compared with the MCTD-non-PAH group (median, 180.0 vs. 57.3 pg/mL, *P* < 0.001). The values of PASP were not routinely tested in the MCTD-non-PAH group, and the data could not be compared. When PAH was diagnosed, nearly half (45.7%) of patients were in WHO Fc III/IV. Meanwhile, only one (1.4%) patient in the MCTD-non-PAH group reached WHO Fc III/IV.

### Factors associated with PAH

**Fig. 1:**
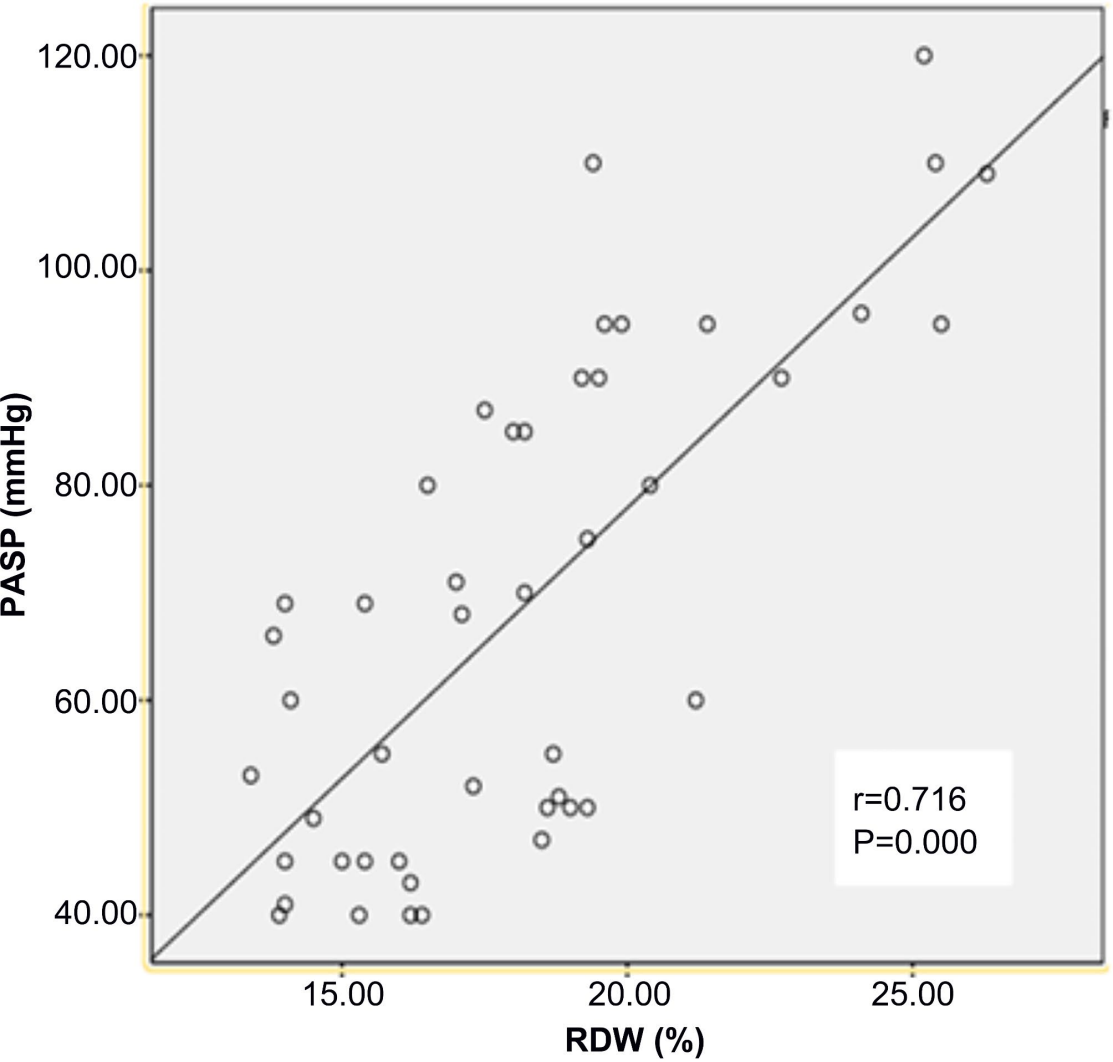
Association between RDW and PASP. Spearman correlations analysis showed a significant positive correlation between RDW and the PASP (r = 0.716, *P <* 0.001) in the study population of MCTD-PAH (n = 46)

Univariable and multivariable binary logistic regression analyses were performed to evaluate the clinical, laboratory, and echocardiographic factors independently associated with PAH. As shown in Table 3, only RDW (OR: 2.128; 95% confidence interval: 1.497–3.026; *P <* 0.001) was significant. Consistently, there was a positive correlation between RDW and PASP (r = 0.716, *P <* 0.001) by Spearman correlations analysis (Fig. 1).

**Table 3:** Multivariable binary logistic regression analysis of statistically significant candidates

	Univariable binary logistic regression analysis				Multivariable binary logistic regression analysis			
Variable	*P*	OR	95% CI		*P*	OR	95% CI	
			Lower limit	Upper limit			Lower limit	Upper limit
RDW	*<* 0.001	2.199	1.663	2.906	*<* 0.001	2.128	1.497	3.026
BNP	*<* 0.001	1.005	1.002	1.009	0.324	1.002	0.998	1.005
UA	0.024	1.004	1.001	1.007	0.791	1.001	0.995	1.006
TSH	0.379	1.055	0.936	1.189				
FT3	0.009	0.624	0.437	0.891	0.280	0.737	0.424	1.282
LVEF	0.445	0.980	0.932	1.031				
LVDd	0.002	0.219	0.084	0.571	0.341	1.980	0.485	8.073
PA	0.025	0.030	0.001	0.646	0.389	0.138	0.002	12.428
LAD	0.142	0.608	0.313	1.182				
Pericardial effusion	0.052	0.441	0.193	1.007	0.854	0.878	0.219	3.518
Chest tightness	< 0.001	0.154	0.067	0.355	0.095	0.169	0.021	1.366
Serositis	< 0.001	0.221	0.100	0.487	0.593	1.736	0.230	13.090

OR, odds ratio; CI, confidential intervals; RDW, red cell distribution width; UA, uric acid; TSH, thyroid stimulating hormone; FT3, free triiodothymnine; LVEF, left ventricle ejection fraction; LVDd, left ventricular diastolic dimension; PA, pulmonary artery velocity; LAD, left atrial diameter

**Fig. 2:**
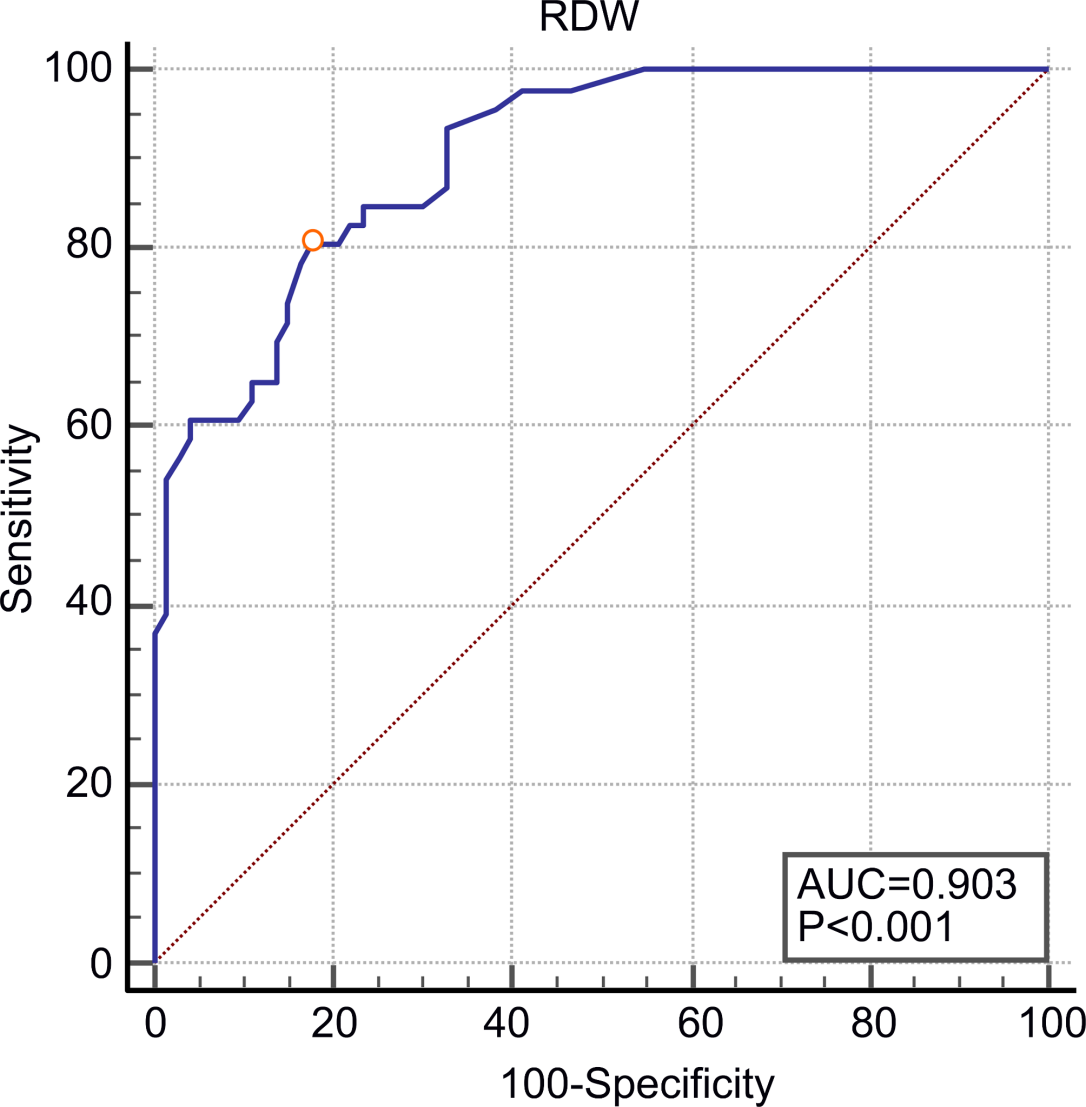
The ROC curve of diagnostic accuracy with RDW for predicting the presence of PAH in MCTD patients. An RDW value > 15.2% was 80.4% sensitive and 82.2% specific for diagnosing PAH, with an AUC of 0.9 (95% confidence interval: 0.835–0.949; *P <* 0.001). Youden index was 0.6

Next, we analyzed the ROC curve of the diagnostic accuracy for RDW (Fig. 2). Using 15.2% as a cutoff level, the RDW value had 80.4% sensitivity and 82.2% specificity for PAH diagnosis. The AUC estimate for RDW was 0.9 (*P <* 0.001), with a Youden index of 0.6.

### Survival

Nineteen out of 119 patients (16.0%) died during a mean follow-up period of 80 ± 38 months. Eight patients were lost to follow-up. One patient committed suicide, and one died in a car accident. The survival rate was higher in the MCTD-non-PAH group compared with the MCTD-PAH group (log-rank *P* < 0.001) (Supplementary Figure S1). Elevated RDW values were associated with an increased risk of all-cause death. Fourteen out of 46 patients (30.4%) died during a mean follow-up period of 68 ± 40 months. Two patients were lost to follow-up. One patient committed suicide. According to the Kaplan-Meier survival analysis, a significant difference was found between the two groups(patients with RDW > 15.2% and patients with RDW ≤ 15.2%) in terms of survival rate (log-rank *P* = 0.049) (Supplementary Figure S2).

## Discussion

CTD-PAH constitutes the second most common subtype of PAH [10], most commonly associated with SSc, SLE, and MCTD [11]. While SSc and SLE are the leading cause of CTD-PAH [12], little is known about MCTD-PAH, despite reports showing that MCTD-PAH was more frequent than SSc and SLE. In this study, PAH diagnosis according to TTE was 1.0 ± 1.6 years’ delayed from PAH onset, accompanied by a significantly higher percentage of WHO Fc III or IV in the MCTD-PAH group compared with the controls (45.7% vs. 1.4%). RDW was the only factor significantly associated with the development of PAH in MCTD patients through multivariable binary logistic regression analysis. Consistent with the literature [13], sex, age, and disease duration were unrelated to PAH.

The first line of diagnostic testing for patients with suspected PAH involves undergoing TTE. Previous reports showed that noninvasive echocardiography had high specificity and sensitivity in detecting PAH when above 36 mmHg [14]. The median PASP by echocardiography of the MCTD-PAH patients was 67.0 mmHg, greatly higher than the diagnostic critical value.

On the other hand, significantly higher pericardial effusion values and lower indexes of LVDd, PA, LAD, and LVEF were also observed in MCTD-PAH patients. Thus, it is likely that the pathophysiology of PAH involves remodeling not only the right heart but also the left heart. In addition, in the present study, 17 of 46 patients (37.0%) had pericardial effusion by echocardiography. The literature suggests that pericardial effusion relates to RV failure and immunologically mediated inflammatory conditions [15]. Pericardial effusion is an increasingly recognized risk factor of PAH related to CTD [16].

Several biomarkers have been recommended to assess the presence and prognosis of PAH [17]. Nagaya et al. reported that PAH patients with BNP > 150 pg/mL at baseline and > 180 pg/mL after therapy had a poor prognosis [18]. The median plasma levels of BNP in the MCTD-PAH group was 180.0 pg/mL, suggesting these patients were in poor condition. UA [19], thyroid dysfunction [20], and the presence of specific autoantibodies [21] were also proposed to be related to PAH. However, the risk of both UA and thyroid dysfunction has not been confirmed in this study, and autoantibody profiles were not significantly different between the two groups.

RDW is an index of blood routine [22], which has been suggested as a biomarker of PAH in patients with other CTD [23, 24]. To our knowledge, this is the first study to identify the importance of RDW in PAH related to MCTD. The sensitivity and specificity of RDW value > 15.2% were 80.4% and 82.2%, respectively, for the prediction of MCTD-PAH. Given its positive correlation with the PASP, RDW may significantly affect the progression of PAH associated with MCTD. Recently, raised RDW values are related to the mortality of PAH and proposed as a prognostic biomarker of poor prognosis in PAH with various etiologies [23, 25], which was even superior to pro-BNP [26]. Xi et al. [27] suggested that RDW independently predicted the responsiveness of acute pulmonary vasodilator testing in idiopathic PAH patients.

The exact pathologic mechanism linking RDW to PAH is still unclear but may be related to vascular injury [28]. Routinely, the most common cause of an increased RDW is anemia related to ineffective erythropoiesis [29]. In the present study, all patients had normal hemoglobin levels with a normal range of MCV in the whole group. There was no iron deficiency; as such, the influence of iron was excluded. It was hypothesized that RDW was involved in inflammation [30]. The elevation of RDW level could reflect the circulating levels of proinflammatory cytokines, such as tumor necrosis factor (TNF)-α, interleukin (IL)- 1, and IL-6, thus leading to the occurrence of PAH [23, 31, 32]. Further studies focusing on mechanisms correlated with RDW and MCTD-PAH are necessary.

FT3 was significantly decreased in MCTD-PAH patients, while TSH was significantly higher in the MCTD-PAH group. The literature suggests that thyroid dysfunction is associated with PAH [33]. Indeed, thyroid hormone impacts cardiac contractility, cardiac output, and pulmonary and systemic vascular resistance [20, 34], and up to 20% of patients with PAH also have thyroid disease [35]. The present study report reinforces the important observation that TSH is associated with PAH, and a future study of thyroid dysfunction as a potential remediable contributor to mortality in PAH is warranted.

## Limitation of the study

A major limitation of this study is not routinely applying the gold standard test for PAH-right-sided heart catheterization (RHC). As an invasive and expensive operation, RHC was not common consent by most patients. Some patients in the present study were too serious to lie flat. The retrospective nature of the study limited the data to those available in the patient charts.

## Conclusion

MCTD patients with elevated RDW had a high risk for PAH. RDW can be used as a simple index for monitoring PAH in clinical practice.

### Electronic supplementary material

Below is the link to the electronic supplementary material.

Supplementary Material 1

## Data Availability

The datasets used and/or analysed during the current study available from the corresponding author Bo Jiang on reasonable request.

## List of abbreviations

PAH: pulmonary arterial hypertension
MCTD: mixed connective tissue disease
ROC: receiver operating characteristic
PASP: pulmonary arterial systolic pressure
BNP: brain natriuretic peptide
RDW: red cell distribution width
SSc: systemic sclerosis
SLE: systemic lupus erythematosus
PM/DM: polymyositis or dermatomyositis
RA: rheumatoid arthritis
CTD: connective tissue disease
TRV: tricuspid regurgitation velocity
pSS: primary Sjögren’s syndrome
TTE: transthoracic echocardiography
BMI: body mass index
BSA: body surface area
WHO-FC: World Health Organization functional classification
UA: uric acid
CRP: C-reactive protein
ESR: erythrocyte sedimentation rate
MCV: mean corpuscular volume SLEDAI-2 K:Systemic Lupus Erythematosus Disease Activity Index 2000
AUC: area under the curves
FT3: serum free triiodothyronine
TSH: thyroid stimulating hormone
LVDd: left ventricular diastolic dimension
PA: pulmonary artery
LAD: left atrial diameter
LVEF: left ventricle ejection fraction
IVSTd: interventricular septum thickness diastolic
LVPWTd: left ventricular posterior wall thickness at end-diastole
AoD: aorta diameter
LVDs: left ventricular end-systolic dimension
E/A: mitral e peak/mitral a peak
TNF: tumor necrosis factor
IL: interleukin
RHC: right-sided heart catheterization
