# Supplementary material for: Association of red cell distribution width with pulmonary arterial hypertension in patients with mixed connective tissue disease
Source: BMC Pulm Med. 2023 Aug 14;23:299. doi: 10.1186/s12890-023-02597-z (PMC10426200; doi:10.1186/s12890-023-02597-z)
Supplement: Supplementary file 1 — Supplementary Material 1 [file 12890_2023_2597_MOESM1_ESM.pdf]

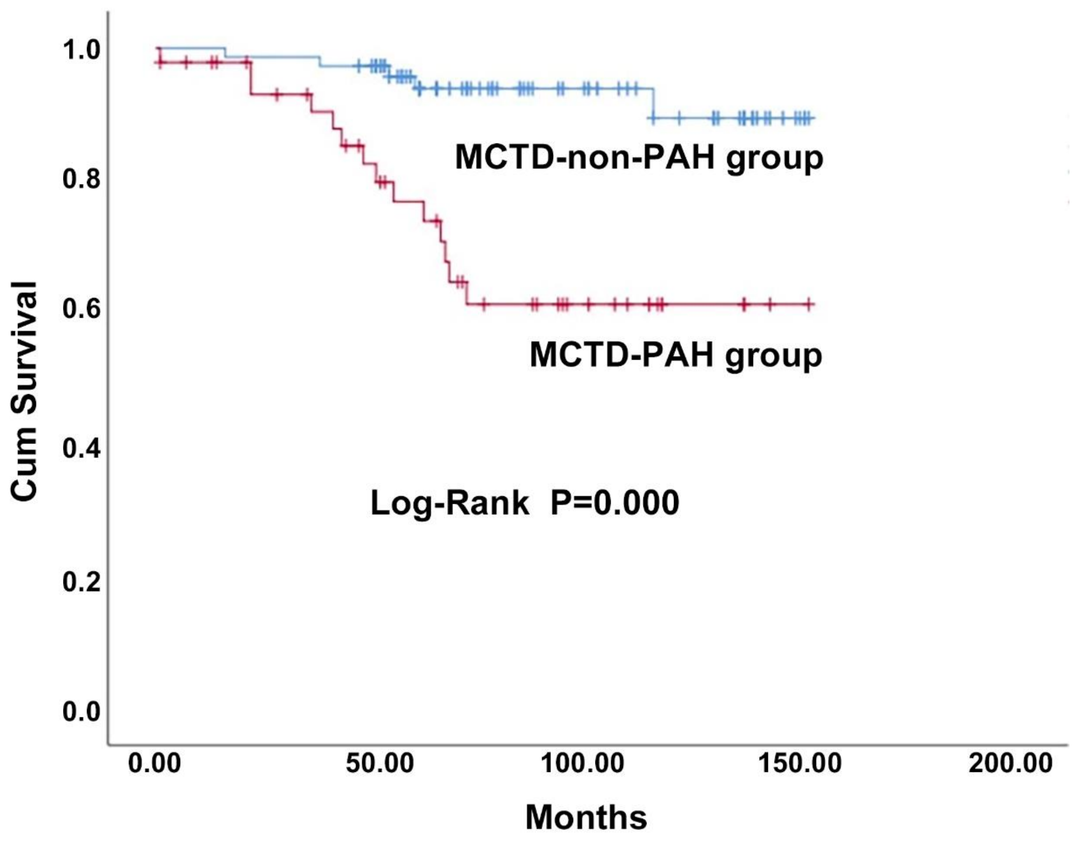

Supplementary Figure S1. Kaplan-Meier survival curves for MCTD-PAH and MCTD-non-PAH patients.

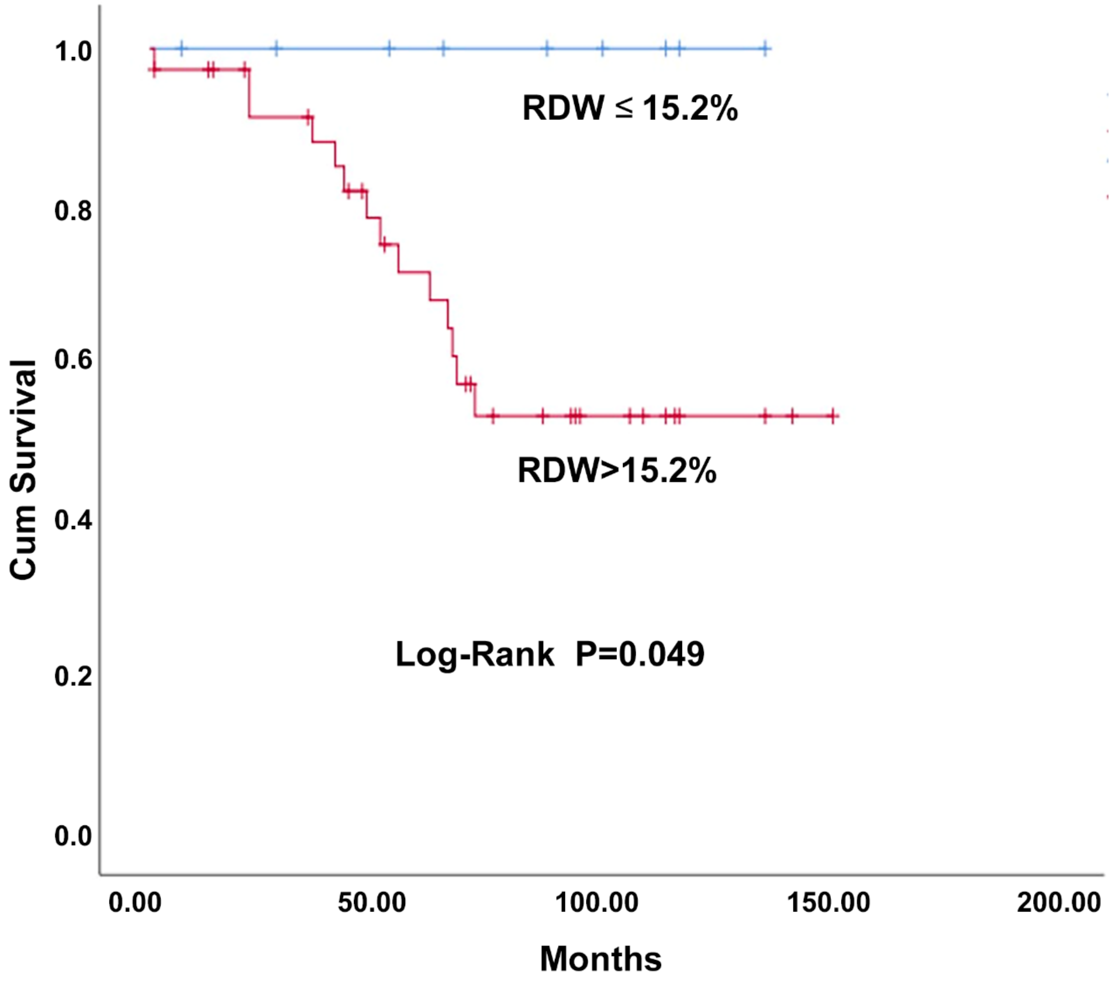

Supplementary Figure S2. Kaplan-Meier survival curves for MCTD-PAH patients stratified by the RDW cutoff value.
